# Two inhibitors of yeast plasma membrane ATPase 1 (*Sc*Pma1p): toward the development of novel antifungal therapies

**DOI:** 10.1186/s13321-018-0261-3

**Published:** 2018-02-20

**Authors:** Sabine Ottilie, Gregory M. Goldgof, Andrea L. Cheung, Jennifer L. Walker, Edgar Vigil, Kenneth E. Allen, Yevgeniya Antonova-Koch, Carolyn W. Slayman, Yo Suzuki, Jacob D. Durrant

**Affiliations:** 1 Division of Host Pathogen Systems and Therapeutics, Department of Pediatrics, School of Medicine, University of California, San Diego, La Jolla, CA 92093 USA; 2 Department of Biological Sciences, University of Pittsburgh, Pittsburgh, PA 15260 USA; 3 Department of Genetics, Yale University School of Medicine, New Haven, CT 06520 USA; 4 Department of Synthetic Biology and Bioenergy, J. Craig Venter Institute, La Jolla, CA 92037 USA

**Keywords:** Antifungal, PMA1, P-type ATPase, Computer modeling, *Saccharomyces cerevisiae*, In vitro evolution, Drug resistance

## Abstract

Given that many antifungal medications are susceptible to evolved resistance, there is a need for novel drugs with unique mechanisms of action. Inhibiting the essential proton pump Pma1p, a P-type ATPase, is a potentially effective therapeutic approach that is orthogonal to existing treatments. We identify NSC11668 and hitachimycin as structurally distinct antifungals that inhibit yeast *Sc*Pma1p. These compounds provide new opportunities for drug discovery aimed at this important target.

**Electronic supplementary material:**

The online version of this article (10.1186/s13321-018-0261-3) contains supplementary material, which is available to authorized users.

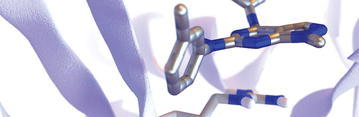

## Background

Antifungal medications are in high demand, but low efficacy, host toxicity, and emerging resistance among clinical strains [1, 2] complicate their use. There is an urgent need for novel antimycotic therapeutics with unique mechanisms of action. The purpose of the current work is to describe two novel antifungals: 4-N,6-N-bis(3-chlorophenyl)-1-methylpyrazolo[3,4-d]pyrimidine-4,6-diamine (NSC11668), and hitachimycin (also known as stubomycin, or NSC343256).

Most antifungals in clinical use target ergosterol, a sterol present in fungal membranes but largely absent from human cells [3]. Polyene antimycotics bind directly to ergosterol, thereby destabilizing the membrane [4]. Allylamines inhibit squalene monooxygenase [5], the first enzyme in the ergosterol biosynthetic pathway [6]. Most azole antifungals inhibit the downstream enzyme lanosterol 14 α-demethylase [7], with the possible exception of abafungin, which may instead affect sterol-C-24-methyltransferase and the fungal cell membrane directly [8].

Only a few approved antimycotics have mechanisms that are unrelated to ergosterol biosynthesis. For example, the highly effective echinocandins inhibit 1,3-β-glucan synthase, hindering production of the critical cell-wall component β-glucan [9, 10]; and the teratogenic compound flucytosine interferes with eukaryotic RNA/DNA synthesis [11, 12]. As these compounds act through pharmacologically distinct mechanisms, they can in principle complement anti-ergosterol interventions (see, for example, Ref. [13]).

Recognizing the need for additional antifungals with mechanisms of action unrelated to ergosterol biosynthesis, we turned our attention to the essential proton pump of the P-type ATPase class (H^+^-ATPase), which is conserved in both plants and fungi [14]. In the model organism *S. cerevisiae*, *Sc*Pma1p generates a proton gradient that is essential for both pH homeostasis and nutrient transport via H^+^-symport. The resulting electrochemical gradient drives further nutrient uptake via uniporters [15]. A *ScPMA1* null mutation is lethal in haploid cells, suggesting that the protein is essential for yeast survival [14].

*Sc*Pma1p inhibitors are not necessarily toxic to humans [16, 17]. A BLASTP search using *Sc*Pma1p as the query sequence (UniProt P05030) revealed that the closest human homologs (calcium-transporting ATPases, e.g., UniProt O75185, A0A0A0MSP0, B7ZA13) share ~ 27% sequence identity. An antimalarial compound known to inhibit *Sc*Pma1p is also advancing through clinical trials, demonstrating that it is possible to develop *Sc*Pma1p-specific small-molecule inhibitors with low host toxicity. *Sc*Pma1p is therefore an attractive target.

*Sc*Pma1p has at least three druggable pockets. The primary, orthosteric pocket binds ATP [18] and decavanadate [19]. A second pocket—which binds the drug digoxin in the homologous Na^+^, K^+^-ATPase [20] —lies between the TM1 and TM4 transmembrane helices [21, 22]. A third, cytoplasm-accessible pocket within the membrane-spanning domain binds spiroindolone [17] and tetrahydrocarbazole [23] inhibitors. Specific binding sites have not yet been validated for most *Sc*Pma1p inhibitors, including DMM-11 [24]; ebselen [25]; the natural products chebulagic acid and tellimagrandin II [26]; and the inhibitors found in a recent high-throughput screen [27]. Other molecules, such as the carbazole inhibitors [28] and demethoxycurcumin [29], do not compete with ATP binding and so must bind elsewhere. Whether these compounds bind the digoxin- or spiroindolone pockets remains unknown. Mutations near both these pockets also confer resistance to omeprazole, a covalent inhibitor with modest activity [30].

We recently discovered that KAE609, an antimalarial compound currently in Phase II clinical trials [16], is cytotoxic to *S. cerevisiae* and inhibits *Sc*Pma1p by binding to the cytoplasm-accessible pocket [17]. As part of a subsequent search for additional, structurally distinct *Sc*Pma1p inhibitors that bind to the same pocket, we used two different experimental assays (vesicular *Sc*Pma1p and whole-cell yeast) to evaluate compounds available from the National Cancer Institute (NCI). These efforts unexpectedly identified two low-micromolar *Sc*Pma1p inhibitors, NSC11668 and hitachimycin, that apparently act via binding to one of the other *Sc*Pma1p pockets. We hypothesize that they target the nucleotide (ATP) binding site rather than the expected cytoplasm-accessible pocket. We hope these leads will be useful in ongoing efforts to identify and optimize novel *Sc*Pma1p inhibitors.

## Results and discussion

### Identifying whole-cell inhibitors

Motivated by the need for novel antimycotics that act orthogonally to existing antiergosterol therapeutics, we first sought to identify chemical compounds with whole-cell biological effects against fungi. *S. cerevisiae* is an excellent model organism representing this kingdom, but its multiple drug efflux pumps often reduce the cytotoxicity of otherwise potent compounds. This yeast defense mechanism can lead researchers to discard molecules that might otherwise be potential leads if chemically optimized and/or coupled with adjuvants to prevent export. We therefore used the ABC_16_-Monster yeast strain, which lacks 16 genes encoding ATP-binding cassette (ABC) transporters [31] and so is more susceptible to cytotoxic compounds.

We experimentally screened the ~ 1500 compounds of the NCI Diversity Set IV, a repository of structurally diverse, freely available small molecules, for activity against a whole-cell ABC_16_-Monster culture. Of the ~ 1500 compounds tested, 36 inhibited yeast growth by at least 97% at 100 μM (Additional file 1: Table S1).

### Confirming *Sc*Pma1p inhibition in a cell-free assay

Using a computational protocol designed to predict small-molecule binding to the cytoplasm-accessible spiroindolone pocket, we selected seven of the whole-cell inhibitors for further study. These compounds were tested for specific activity against *Sc*Pma1p in a vesicle-based (cell-free) assay. In brief, we used a yeast strain that is prone to vesicle production due to an engineered defect in secretory-vesicle/plasma-membrane fusion. We transformed these yeast with a *Sc*PMA1 overexpression plasmid, so the harvested vesicles bore high levels of *Sc*Pma1p. *Sc*Pma1p inhibition was measured by monitoring ATP hydrolysis in the presence of the vesicles. Small-molecule *Sc*Pma1p inhibitors prevented ATP hydrolysis, reducing the measured concentration of inorganic phosphate. See Ref. [17] for full details.

Two active compounds, NSC11668 and hitachimycin, had IC_50_ values of 4.4 μM and 7.8 μM in the cell-free assay, respectively (Additional file 1: Figure S1A). These same compounds had IC_50_ values of 14.8 ± 1.24 (s.e.m) and 0.87 ± 0.11 μM against the whole-cell ABC_16_-Monster strain (see Additional file 1: Figure S2, Rows A and B).

### The compounds are unlikely to bind the cytoplasm-accessible spiroindolone pocket

We tested both *Sc*Pma1p inhibitors (in duplicate) against the unmodified ABC_16_-Monster strain, as well as against two ABC_16_-Monster strains that each contained distinct spiroindolone-pocket *ScPMA1* mutations: L290S and P399T (Additional file 1: Figure S3) [17]. As expected, the IC_50_ values of NSC11668 and hitachimycin against the unmodified ABC_16_-Monster strain were roughly equal to those found previously. As a positive control, we also tested KAE609, a known *Sc*Pma1p inhibitor that binds the cytoplasm-accessible (spiroindolone-binding) pocket. It, too, had an unmodified-strain IC_50_ comparable to that measured previously [17].

The positive control (KAE609) had a higher IC_50_ value when tested against the ABC_16_-Monster strains with spiroindolone-pocket *ScPMA1* mutations. This is expected; the mutations likely disrupt KAE609 binding, thereby reducing its potency. Given that our computational protocol targeted the same *Sc*Pma1p pocket, we expected NSC11668 and hitachimycin IC_50_ values to be similarly mutation dependent. But the IC_50_ values of these inhibitors against the modified and unmodified ABC_16_-Monster strains were roughly the same, suggesting that the compounds do not bind the spiroindolone pocket (Additional file 1: Figure S3).

That having been said, these results cannot entirely rule out spiroindolone-pocket binding. NSC11668 and hitachimycin binding to additional targets may be primarily responsible for growth inhibition, such that spiroindolone-pocket binding, though legitimate, has little biological effect. In the case of hitachimycin, whole-cell inhibition was more potent than cell-free *Sc*Pma1p inhibition, supporting this possibility (Additional file 1: Figures S1A and S2). We note, also, that the point mutations produced only a twofold reduction in the potency of our control compound NITD609, a low-nanomolar spiroindolone-pocket-binding *Sc*Pma1p inhibitor [17] (Additional file 1: Figures S1B and S3C). These issues aside, we nevertheless believe that the most likely explanation for our experimental results is that NSC11668 and hitachimycin do not bind the spiroindolone pocket.

### NSC11668 and hitachimycin are not general, non-specific binders

At sufficiently high concentrations, many small molecules form colloidal aggregates between 100 and 1600 nm across. Protein adsorption to colloidal surfaces can lead to denaturation. Small-molecule aggregation is thus a major cause of non-specific inhibition, often yielding false positives in early-stage drug-discovery campaigns.

#### Nsc11668

We performed a cheminformatics search to verify that NSC11668 does not aggregate. First, given that detergent disrupts colloid formation [32, 33], we searched for examples of detergent-dependent NSC11668 inhibition. If a compound inhibits via aggregation, one would expect inhibition in the absence of detergent that is abolished when detergent is added. Two biochemical assays catalogued in PubChem [34, 35] (AIDs 584 and 585) tested for AmpC beta-lactamase inhibition in the presence and absence of the detergent Triton X-100 [32], respectively. NSC11668 did not inhibit AmpC beta-lactamase in either screen, demonstrating that it does not aggregate.

In another screen (AID 624002), NSC11668 inhibited mutant isocitrate dehydrogenase 1 in the low-micromolar range, even in the presence of the detergent TWEEN 20. Similar screens against glucocerebrosidase-p2 (AID 348) and the thioesterase domain of fatty acid synthase (AID 602261) have shown NSC11668 activity in the presence of detergent. Again, if NSC11668 were a general, non-specific inhibitor/aggregator, the detergent should have disrupted any colloid formation. These screens similarly demonstrate that NSC11668 is capable of specific inhibition.

To further rule out the possibility of general, non-specific inhibition, we considered all 794 PubChem-catalogued screens that included NSC11668 (August, 2017). To identify screens that used cell-free assays to measure NSC11668 activity against specific proteins, we (1) discarded the screens that did not include a listed target; (2) retained only screens that explicitly listed NSC11668 as active or inactive; (3) and removed screens that contained words in their titles or protocol descriptions that suggested a cell-based assay (e.g., “cell-based,” “cell line,” “cell suspension,” etc.). NSC11668 inhibited only two of the 217 unique proteins that remained. Inhibition of a third protein was inconclusive because different screens yielded different results. The total hit rate, then, is somewhere between 0.9 and 1.4%. We would expect this rate to be higher if non-specific inhibition were at play.

NSC11668 is thus not a general, non-specific inhibitor, but it does appear to be promiscuous. Further optimization will be required to achieve P-type ATPase specificity. Aside from the targets identified in the cell-free assays above, cell-based assays suggest inhibition of the TIM22 import pathway, the GLP-1 receptor, HSP90, and tyrosyl-DNA phosphodiesterase 1 (see AIDs 493003, 540268, 540270, 624417, 686978 and 686979). In some cases, NSC11668 polypharmacology may be beneficial. For example, NSC11668 is also known to inhibit *H. sapiens* ABCG2 [36], an ABC transporter like those that are deleted in the ABC_16_-Monster strain. ABCG2 plays roles in transporting both xenobiotics [37, 38] and diverse endogenous molecules, ranging from heme [39, 40] to urate [41] to riboflavin [38]. NSC11668 may therefore inhibit its own cellular export, potentiating any pharmacological effect.

#### Hitachimycin

Cheminformatics analyses also suggest hitachimycin is capable of specific inhibition. Hitachimycin is active in three PubChem-catalogued assays that include aggregation-preventing detergent: AID 652105, against phosphatidylinositol 5-phosphate 4-kinase in the presence of the detergent CHAPS; and AIDs 1053136 and 743269, against HIV-1 LEDGF/p75 DNA integration in the presence of the detergent Brij-35. The compound has been studied previously as a possible antibiotic, antifungal, and antitumor agent [42, 43].

A total of 226 screens tested hitachimycin for biological activity. Applying the same filters described above, we identified 55 potential protein targets. Of these, hitachimycin was active against only three (5.5%). We would again expect this rate to be higher if hitachimycin acted via non-specific inhibition.

### NSC11668 and hitachimycin may bind the *Sc*Pma1p ATP-binding pocket

NSC11668 and hitachimycin inhibit *Sc*Pma1p, but they do not bind the *Sc*Pma1p spiroindolone pocket. We therefore hypothesize that they bind the *Sc*Pma1p ATP-binding pocket, similar to the general ATPase inhibitor decavanadate [19]. Our whole-cell and cell-free (vesicle-based) assays cannot distinguish between ligand binding to the different *Sc*Pma1p sites, so we turned to homology modeling and computer docking. We acknowledge that these computational techniques are only predictive, but they can be useful tools for hypothesis generation.

#### Homology modeling

We used a *Sc*Pma1p homology model described in detail elsewhere [17]. Homology modeling was critical for this project because, as a large transmembrane protein, *Sc*Pma1p has been difficult to isolate and crystallize. To create the *Sc*Pma1p model, we used the Na^+^, K^+^-ATPase from *Sus scrofa* as a structural template (PDB 3N2F, chain C) [20]. Per a Clustal Omega alignment [44, 45], these two proteins share 27.5% sequence identity (see UniProt P05030 and P05024, respectively) [46].

The amino acids that form the ATP binding site are even more conserved across the two proteins. We examined an ADP-bound *Sus scrofa* Na^+^, K^+^-ATPase structure (3WGU:A) [47] and identified 19 pocket-lining amino acids that come within 4 Å of the crystallographic ADP molecule: T371, D443, S445, E446, F475, S477, K480, Q482, K501, G502, A503, R544, V545, L546, T610, G611, D612, R685, and N713. Thirteen (68%) of these were identical in the two species (*S. scrofa* vs. *S. cerevisiae)*. Additionally, both proteins have negatively charged amino acids at residue 446 (glutamic acid vs. aspartic acid, respectively) and hydrophobic side chains at residue 545 (valine vs. alanine, respectively). These ATP-binding-pocket similarities help justify our use of 3N2F:C as a structural template.

The 3N2F-based model captures *Sc*Pma1p in the E2P (cation-free) conformation. In this conformation, Mg^2+^ alone is bound, and the cation is accessible to the extracellular space for export. We also modeled *Sc*Pma1p in the E1P (cation-bound) conformation using a different *Sus scrofa* Na^+^,K^+^-ATPase structure as the template (PDBID: 3WGV) [47]. While this second conformation may prove useful in future ligand-discovery efforts, we opted not to pursue it in the present study. Docking the *Sc*Pma1p inhibitors NITD609 and NITD246 into the spiroindolone binding pocket of the 3WGV-based model gave seemingly implausible poses. We were therefore reluctant to pursue the E1P model further.

#### Computer docking

To generate binding-mode hypotheses, we used AutoDock Vina [48] to dock NSC11668 and hitachimycin into the ATP binding site of our 3N2F-based (E2P) homology model. We considered the top-scoring Vina pose for each docked compound.

**Fig. 1:**
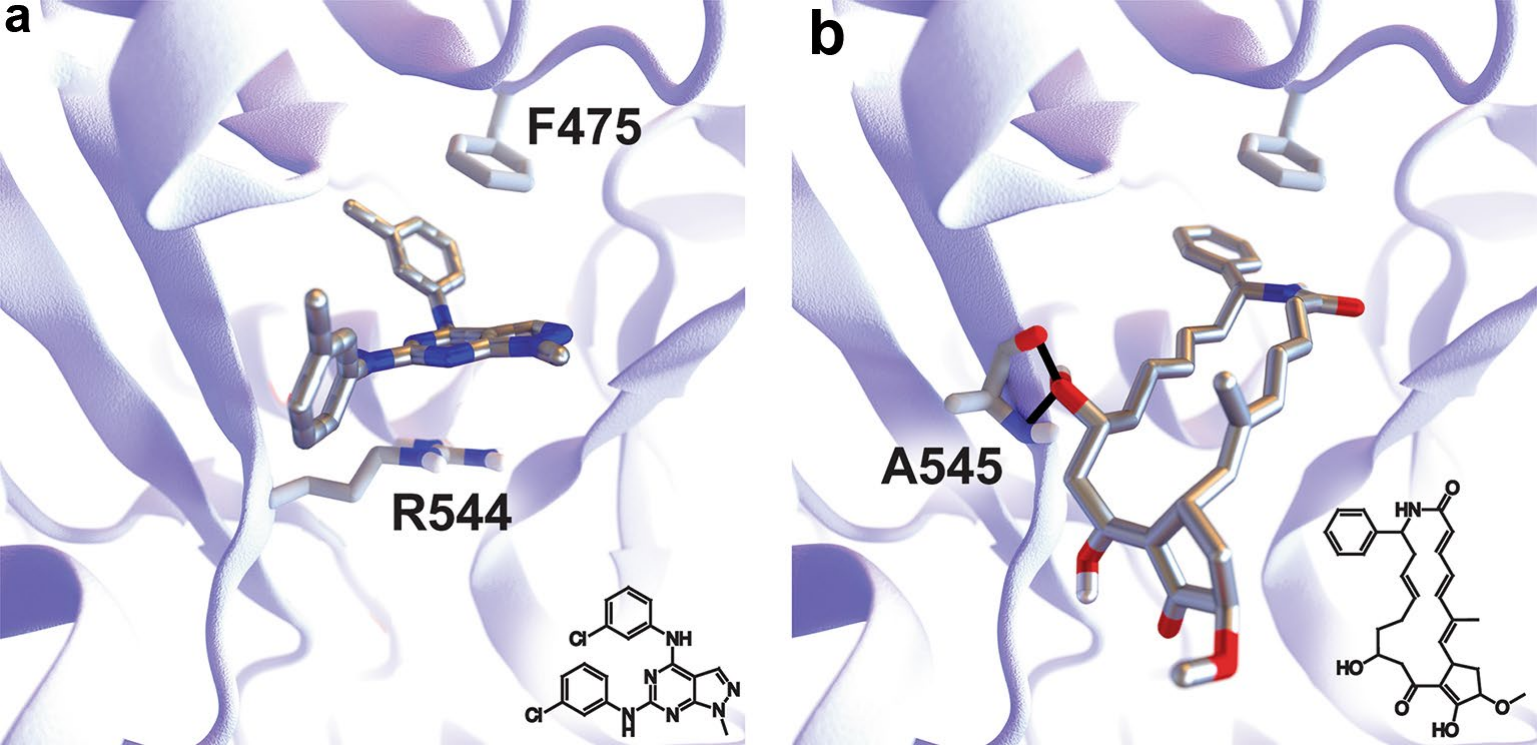
Predicting binding poses. **a** NSC11668 is predicted to bind the ATP-binding pocket. Its central 1*H*-pyrazolo[3,4-*d*]pyrimidine moiety may form a cation-π interaction with R544. One if its chlorobenzene moieties may form a π–π stacking interaction with F475, as does ADP in the 3WGU structure. **b** Hitachimycin is similarly predicted to bind the ATP-binding pocket. One of its hydroxyl moieties may hydrogen bond with the A545 backbone. Its benzene moiety may also form a π–π stacking interaction with F475

Figure 1a illustrates the predicted molecular interactions between NSC11668 and the *Sc*Pma1p ATP-binding pocket. The central 1*H*-pyrazolo[3,4-*d*]pyrimidine moiety forms a cation-π interaction with R544, and one if the chlorobenzene moieties forms a π–π stacking interaction with F475. This second interaction is notable because the ATP adenine moiety forms a similar π–π stacking interaction with F475, per the 3WGU structure [47]. Figure 1b illustrates the predicted molecular interactions between hitachimycin and the ATP-binding pocket. A compound hydroxyl moiety hydrogen bonds with the A545 backbone, and the benzene moiety also forms a π–π stacking interaction with F475.

### Follow-up whole-cell studies

We ultimately chose not to pursue hitachimycin further as a drug lead. LC–MS analysis determined that the hitachimycin sample we obtained from the NCI was only 67.3% pure, and the ZINC [49] database identified no additional vendors. Hitachimycin was also absent from the MolPort database, which includes compounds that are commercially available through many suppliers. We therefore cannot rule out the possibility that a sample impurity inhibits *Sc*Pma1p rather than hitachimycin itself.

In contrast, LC–MS analysis revealed that the NSC11668 sample obtained from the NCI was 95.9% pure. We therefore considered NSC11668 to be the more promising lead. NSC11668 satisfies all of Lipinski’s rules for drug-like molecules [50, 51] according to Schrodinger’s QikProp software [52] (molecular weight: 385.255; hydrogen-bond donors: 2; hydrogen-bond acceptors: 4; predicted LogP: 4.891). NSC11668 possesses a rigid, purine-like scaffold similar to that of many other pharmaceuticals. Indeed, bicyclic compounds with pyrimidine-diamine substructures, e.g., olomoucine [53], H717 [54], and seliciclib [55], are currently being pursued as possible cyclin-dependent kinase inhibitors.

To further explore the pharmacological potential of NSC11668, we verified its activity against whole-cell, wild-type *S. cerevisiae*. The compound is active in the low-micromolar range even when *S. cerevisiae*’s drug efflux pumps are intact (IC_50_ of 20.3 ± 4.18 μM, see Additional file 1: Figure S2, Row C).

We also evaluated NSC11668 for human cellular cytotoxicity. NSC11668 had an IC_50_ of 22.67 ± 2.77 μM against human hepatocarcinoma HepG2. To develop NSC11668 into an orally available drug, further optimization will be required to improve the therapeutic index. However, regardless of the potency of this specific compound, NSC11668 represents a new *Sc*Pma1p-inhibiting scaffold class that will prove useful in future drug-discovery projects. We also note that its IC_50_ value is comparable to that of other clinically approved antifungals tested in our yeast model (e.g., topical ciclopirox: ~ 30 μM; oral fluconazole: ~ 3 μM; oral itraconazole: ~ 5 μM; topical miconazole: ~ 3 μM). NSC11668 therefore warrants continued study.

## Conclusions

In summary, we have identified NSC11668 and hitachimycin as antifungal molecules that target *Sc*Pma1p, a transmembrane protein crucial for pH homeostasis in fungal pathogens [14]. We judge NSC11668 to be the more promising drug lead.

As expected, NSC11668 showed less efficacy against wild-type yeast than against the ABC_16_-Monster strain, highlighting the strength of the ABC_16_-Monster technique as a tool for identifying novel molecules and targets. Phenotypic screens against ABC_16_-Monster, which lacks 16 drug efflux pumps that might otherwise reduce intracellular inhibitor concentrations, may identify potential drug leads that would be missed in wild-type screens. NSC11668 binds to *Sc*Pma1p with low-micromolar affinity and is therefore a candidate for chemical optimization. Even slight modifications to compounds can sometimes drastically improve efficacy. KAE261, a low-micromolar *Sc*Pma1p inhibitor that binds the spiroindolone-binding pocket, is one of many examples that could be cited. KAE585, which differs from KAE261 only by the addition of a single halide atom, is 100 times more potent against *Sc*Pma1p (EC_50_ = ~ 100 nM) than is KAE261 [17].

An obvious future direction is to improve the potency of these compounds against wild-type yeast. Simple molecular modifications, especially those aimed at reducing hydrophobicity [56], can often reduce efflux [57, 58]. Cancer drug discovery provides several excellent examples of this approach. Human ABC transporters such as P-gp and MRP1 contribute to chemotherapy resistance, but small modifications to anthracycline compounds produce compounds such as annamycin with reduced export, leading to substantially improved activity against multidrug-resistant cancer cells [59]. Peptide-conjugated doxorubicin is also effective against doxorubicin-resistant cells for the same reason [60]. We therefore believe that carefully considered medicinal chemistry may reduce NSC11668 efflux as well.

Direct inhibition of ABC transporters is another promising approach. Holmes et al. [61] recently used clorgyline, an inhibitor of fungal ABC and MFS efflux pumps, to reverse azole resistance in yeast. Similarly, Schuetzer-Muehlbauer et al. showed that several ABC-transporter inhibitors, including terbinafine, propafenones, FK506, and GP382, may also function as fungal “chemosensitizers [62],” as may baicalein [63].

This efflux-inhibitor approach has also been validated in other contexts. Verapamil, an L-type calcium channel blocker already in clinical use, has been shown to reduce vincristine resistance in cancer cells by blocking ABC transporters [64]. Other cancer chemosensitizers have also been described [65–67]. Similarly, some have hypothesized that ZnO nanoparticles may enhance the activity of ciprofloxacin against *S. aureus* via inhibition of the NorA efflux protein [68]. It is interesting that NSC11668 is itself an ABCG2 inhibitor [36], suggesting that it may sensitize cells to its own *Sc*Pma1p activity. Even if this sensitization is limited, administering NSC11668 with an efflux-inhibiting adjuvant may still be a promising approach for antifungal therapy.

## Methods

### Yeast strains

Control strain (strain name SY025) = *S. cerevisiae*. Genotype: *MAT***a** *ho∆::[tetO*_*2*_*pr*-*GFP, URA3] can1∆::*GMToolkit-**a** *lyp1∆ his3∆1 leu2∆0 ura3∆0 met15∆0. PMA L290S and PMA1 P339T have been described previously* [17].

ABC_16_-Monster = *S. cerevisiae* Genotype: *MATa adp1*∆ *snq2*∆ *ycf1*∆ *pdr15*∆ *yor1*∆ *vmr1*∆ *pdr11*∆ *nft1*∆ *bpt1*∆ *ybt1*∆ *ynr070w*∆ *yol075c*∆ *aus1*∆ *pdr5*∆ *pdr10*∆ *pdr12can1∆::*GMToolkit-**a** *lyp1∆ his3∆1 leu2∆0 ura3∆0 met15∆0* (deletions for the ABC transporter genes are marked with *[tetO*_*2*_*pr*-*GFP, URA3]*).

### Whole-cell yeast assays

For all yeast growth assays, *S. cerevisiae* was obtained from frozen stocks. Cultures were established using cells taken from single colonies grown on agar plates and inoculated into 2 mL of YPD in 5 mL snap-cap culture tubes. The tubes were grown overnight at 250 RPM in a shaking incubator at 30 °C (Controlled Environment Incubator Shaker, Model G-25, New Brunswick Scientific Co., Inc.). Cultures were extracted during the mid-log growth phase, as judged by an OD600 (600 nm) reading between 0.1 and 0.5. The cells were then diluted in YPD to OD600 0.1 and then again 10× in YPD for a final OD600 of 0.01.

For the whole-cell sensitivity assay, cells at OD600 0.01 were plated onto a 96-well plate with a volume of 100 μL. The compounds of the NCI Diversity Set IV were transferred using a 96 Pin Replicator (Thermo Scientific Nunc) that had been sterilized with 70% ethanol and flamed with a Bunsen burner. The final NCI-compound concentration was 100 μM. The replicator was submerged in DI water, stamped with the NCI Diversity Set IV plate, and released into a 96-well plate with ABC_16_-Monster cells. After an initial reading of OD600 using a Synergy HT spectrophotometer, plates were covered with a lid and placed in an incubator at 30 °C for 18 h. Following incubation, the plates were shaken for 1 min on the “high” setting and immediately read at OD600. Sensitivity was determined by comparing growth relative to a DMSO control.

For the IC_50_ assays, cells at OD600 0.01 were transferred to a 96-well plate (final OD600 = 0.01). At least three independent biological replicates of technical duplicates were used to calculate the IC_50_ in each experiment. Eight two-fold serial dilutions were performed with a top concentration of 150 μM. After an initial reading of OD600 using a Synergy HT spectrophotometer, plates were covered with a lid and placed in an incubator at 30 °C for 18 h. Following incubation, the plates were shaken for 1 min on the “high” setting and immediately read at OD600.

OD600 values at time 0 (h) were subtracted from OD600 values at time 18 h. Nonlinear regression on log(inhibitor) versus response with variable slope (four parameters) was performed using Graphpad Prism, which determined the IC_50_ value for each pair of technical duplicates, with minimum values constrained to 0.0. These IC_50_ values were then averaged across each of the biological replicates.

### *Sc*Pma1p ATPase assay

In this vesicle-based assay, ATP hydrolysis was assayed at 30 °C in 0.5 mL of an ATP regenerating system (5 mM phosphoenolpyruvate and 50 μg/mL pyruvate kinase), 50 mM MES/Tris pH 6.25, 5 mM NaN_3_, 5 mM Na_2_ ATP (Roche), and 10 mM MgCl_2_. Fiske and Subbarow reagent [69] was used to terminate the reaction after 20 min. Following an additional 45 min of color development, the release of inorganic phosphate was measured as absorption at 660 nm. Full details can be found in Ref. [17].

### HepG2 cytotoxicity assay

HepG2-A16-CD81EGFP, i.e., human hepatocarcinoma HepG2 cells stably transformed to express the tetraspanin CD81 receptor [70, 71], were cultured at 37 °C in 5% CO_2_ in DMEM (Life Technologies, CA) supplemented with 10% FBS, 0.29 mg/ml glutamine, 100 unit penicillin, and 100 μg/mL streptomycin. For the HepG2 cytotoxicity assays, 3 × 10^3^ of the HepG2-A16-CD81EGFP cells in 5 μl of assay medium (DMEM without Phenol Red, 5% FBS, and 5x Pen Strep Glutamine; Life Technologies, CA) at 6 × 10^5^ cells/ml were seeded in 1536-well plates (Greiner BioOne white solid bottom custom GNF mold). Compounds were prepared in 12-point 1:3 serial dilutions in DMSO, with the top concentration starting at 10 mM. 50 nl of the compounds in DMSO (0.5% final DMSO concentration per well) were transferred with Acoustic Transfer System (ATS) (Biosero) into the assay plates. Puromycin (12-point serial dilution starting at 10 μM) and 0.5% DMSO were used as positive and negative controls, respectively. After incubation at 37 °C for 72 h, HepG2 cytotoxicity was assessed by removing the media via an inverted spin of the plates at 150 g for 30 s, followed by the addition of 2 μL CellTiterGlo reagent (Promega diluted 1:2 with deionized water) per well using the MicroFlo liquid handler (BioTek). Immediately after the addition of the luminescence reagent, the plates were vortexed for 10 s and read with an EnVision Multilabel reader (PerkinElmer). IC_50_ values were obtained using the normalized bioluminescence intensity and a non-linear variable-slope four-parameter regression curve-fitting model in Prism 6 (GraphPad Software Inc).

### Homology modeling

The homology model has been described in detail elsewhere [17]. In brief, the model was built with Schrödinger’s Prime software [72] using the UniProt [46] sequence P05030 and a structure of a homologous sodium–potassium pump from *Sus scrofa* (PDBID: 3N2F, chain C) [20]. Schrödinger’s knowledge-based method was used, followed by refinement with the Protein Preparation Wizard [73]. The resulting PDB file was converted to AutoDock Vina’s PDBQT format using AutoDockTools [74].

### Virtual screening

Three-dimensional small-molecule models of NSC11668 and hitachimycin (NSC343256) were prepared using Schrodinger’s LigPrep module. Epik [75] assigned protonation states at pH values ranging from 5.0 to 9.0. No more than one low-energy ring conformation was selected for each compound, alternate tautomeric states were considered, and all chiralities were varied except for those specified in the initial structures, allowing at most 32 variations per molecule. Geometries were relaxed using the OPLS_2005 forcefield [76, 77]. The resulting SDF models were converted to AutoDock Vina’s PDBQT format using Open Babel [78] and AutoDockTools [74].

NSC11668 and hitachimycin were docked into *Sc*Pma1p using AutoDock Vina [17]. The docking box measured 30 Å × 30 Å × 30 Å, centered on the ATP-binding pocket. The default Vina “exhaustiveness” parameter was used (eight).

## Additional file

**Additional file 1: Table S1.** A list of additional antifungal compounds found in our whole-cell screen. **Figure S1.** IC_50_ curves for the cell-free, vesicle-based *Sc*Pma1p assays. **Figure S2.** IC_50_ curves for the whole-cell assays. **Figure S3.** Compound IC_50_ values against whole-cell ABC_16_-Monster yeast, with and without two distinct spiroindolone-binding-pocket *Sc*PMA1 mutations (L290S and P399T).

## Abbreviations

ABC_16_-Monster yeast strain: a yeast strain that lacks 16 genes encoding ATP-binding cassette (ABC) transporters
EC_50_: half maximal effective concentration
IC_50_: half maximal inhibitory concentration
NCI: National Cancer Institute
OD600: optical density measured at a wavelength of 600 nm
*S. cerevisiae*: *Saccharomyces cerevisiae*, a model organism also known as baker’s yeast
*Sc*Pma1p: the plasma membrane ATPase 1 protein from yeast
*ScPMA1*: the gene that encodes *Sc*Pma1p

## References

[1] Kathiravan MK, Salake AB, Chothe AS, Dudhe PB, Watode RP, Mukta MS (2012). The biology and chemistry of antifungal agents: a review. Bioorg Med Chem.

[2] Persidis A (1999). Antibacterial and antifungal drug discovery. Nat Biotechnol.

[3] Dupont S, Lemetais G, Ferreira T, Cayot P, Gervais P, Beney L (2012). Ergosterol biosynthesis: a fungal pathway for life on land?. Evolution.

[4] Hamilton JM (1973). Chemistry and biology of polyene macrolide antibiotics. Bacteriol Rev..

[5] Ryder NS (1992). Mechanism of action of allylamine antifungal drugs. Recent Prog Antifung Chemother.

[6] Parveen M, Hasan MK, Takahashi J, Murata Y, Kitagawa E, Kodama O (2004). Response of Saccharomyces cerevisiae to a monoterpene: evaluation of antifungal potential by DNA microarray analysis. J Antimicrob Chemother.

[7] Becher R, Wirsel SGR (2012). Fungal cytochrome P450 sterol 14 alpha-demethylase (CYP51) and azole resistance in plant and human pathogens. Appl Microbiol Biotechnol.

[8] Borelli C, Schaller M, Niewerth M, Nocker K, Baasner B, Berg D (2008). Modes of action of the new arylguanidine abafungin beyond interference with ergosterol biosynthesis and in vitro activity against medically important fungi. Chemotherapy.

[9] Morris MI, Villmann M (2006). Echinocandins in the management of invasive fungal infections, part 1. Am J Health Syst Pharm.

[10] Morris MI, Villmann M (2006). Echinocandins in the management of invasive fungal infections, part 2. Am J Health Syst Pharm.

[11] Vermes A, Guchelaar HJ, Dankert J (2000). Flucytosine: a review of its pharmacology, clinical indications, pharmacokinetics, toxicity and drug interactions. J Antimicrob Chemother.

[12] King CT, Rogers PD, Cleary JD, Chapman SW (1998). Antifungal therapy during pregnancy. Clin Infect Dis.

[13] Petraitis V, Petraitiene R, Sarafandi AA, Keleher AM, Lyman CA, Casler HE (2003). Combination therapy in treatment of experimental pulmonary aspergillosis: synergistic interaction between an antifungal triazole and an echinocandin. J Infect Dis.

[14] Serrano R, Kiellandbrandt MC, Fink GR (1986). Yeast plasma-membrane ATPase is essential for growth and has homology with (Na ++K +), K + - and Ca-2 + -ATPases. Nature.

[15] Sondergaard TE, Schulz A, Palmgren MG (2004). Energization of transport processes in plants. Roles of the plasma membrane H + -ATPase. Plant Physiol.

[16] White NJ, Pukrittayakamee S, Phyo AP, Rueangweerayut R, Nosten F, Jittamala P (2014). Spiroindolone KAE609 for falciparum and vivax malaria. N Engl J Med.

[17] Goldgof GM, Durrant JD, Ottilie S, Vigil E, Allen KE, Gunawan F (2016). Comparative chemical genomics reveal that the spiroindolone antimalarial KAE609 (Cipargamin) is a P-type ATPase inhibitor. Sci Rep..

[18] Ambesi A, Miranda M, Petrov VV, Slayman CW (2000). Biogenesis and function of the yeast plasma-membrane H(+)-ATPase. J Exp Biol.

[19] Clausen JD, Bublitz M, Arnou B, Olesen C, Andersen JP, Moller JV (2016). Crystal structure of the vanadate-inhibited Ca(2 +)-ATPase. Structure.

[20] Laursen M, Gregersen JL, Yatime L, Nissen P, Fedosova NU (2015). Structures and characterization of digoxin- and bufalin-bound Na + , K + -ATPase compared with the ouabain-bound complex. Proc Natl Acad Sci USA.

[21] Obara K, Miyashita N, Xu C, Toyoshima L, Sugita Y, Inesi G (2005). Structural role of countertransport revealed in Ca2 + pump crystal structure in the absence of Ca2+. Proc Natl Acad Sci USA.

[22] Laursen M, Bublitz M, Moncoq K, Olesen C, Moller JV, Young HS (2009). Cyclopiazonic acid is complexed to a divalent metal ion when bound to the sarcoplasmic reticulum Ca2 + -ATPase. J Biol Chem.

[23] Bublitz M, Kjellerup L, Cohrt KO, Gordon S, Mortensen AL, Clausen JD (2018). Tetrahydrocarbazoles are a novel class of potent P-type ATPase inhibitors with antifungal activity. PLoS ONE.

[24] Witek S, Goffeau A, Nader J, Luczynski J, Lachowicz TM, Kuta B (1997). Lysosomotropic aminoesters act as H + -ATPase inhibitors in yeast. Folia Microbiol.

[25] Billack B, Pietka-Ottlik M, Santoro M, Nicholson S, Mlochowski J, Lau-Cam C (2010). Evaluation of the antifungal and plasma membrane H + -ATPase inhibitory action of ebselen and two ebselen analogs in S-cerevisiae cultures. J Enzym Inhib Med Chem.

[26] Kongstad KT, Wubshet SG, Johannesen A, Kjellerup L, Winther AM, Jager AK (2014). High-resolution screening combined with HPLC-HRMS-SPE-NMR for identification of fungal plasma membrane H(+)-ATPase inhibitors from plants. J Agric Food Chem.

[27] Kjellerup L, Gordon S, Cohrt KO, Brown WD, Fuglsang AT, Winther AML (2017). Identification of antifungal H + -ATPase inhibitors with effect on plasma membrane potential. Antimicrob Agents Chemother..

[28] Clausen JD, Kjellerup L, Cohrt KO, Hansen JB, Dalby-Brown W, Winther AML (2017). Elucidation of antimicrobial activity and mechanism of action by N-substituted carbazole derivatives. Bioorg Med Chem Lett.

[29] Dao TT, Sehgal P, Tung TT, Moller JV, Nielsen J, Palmgren M (2016). Demethoxycurcumin is a potent inhibitor of P-Type ATPases from diverse kingdoms of life. PLoS ONE.

[30] Monk BC, Mason AB, Abramochkin G, Haber JE, Setoyoung D, Perlin DS (1995). The yeast plasma-membrane proton-pumping ATPase is a viable antifungal target. 1. Effects of the cysteine-modifying reagent omeprazole. BBA-Biomembranes.

[31] Suzuki Y, Stam J, Novotny M, Yachie N, Lasken RS, Roth FP (2012) The green monster process for the generation of yeast strains carrying multiple gene deletions. J Vis Exp 70:e4072 10.3791/4072 PMC3575205 23271437

[32] Feng BY, Simeonov A, Jadhav A, Babaoglu K, Inglese J, Shoichet BK (2007). A high-throughput screen for aggregation-based inhibition in a large compound library. J Med Chem.

[33] Irwin JJ, Duan D, Torosyan H, Doak AK, Ziebart KT, Sterling T (2015). An aggregation advisor for ligand discovery. J Med Chem.

[34] Wang Y, Suzek T, Zhang J, Wang J, He S, Cheng TJ (2014). PubChem BioAssay: 2014 update. Nucleic Acids Res.

[35] Wang Y, Xiao J, Suzek TO, Zhang J, Wang J, Bryant SH (2009). PubChem: a public information system for analyzing bioactivities of small molecules. Nucleic Acids Res..

[36] Henrich CJ, Robey RW, Bokesch HR, Bates SE, Shukla S, Ambudkar SV (2007). New inhibitors of ABCG2 identified by high-throughput screening. Mol Cancer Ther.

[37] Zhang W, Mojsilovic-Petrovic J, Andrade MF, Zhang H, Ball M, Stanimirovic DB (2003). The expression and functional characterization of ABCG2 in brain endothelial cells and vessels. FASEB J..

[38] Vlaming MLH, Lagas JS, Schinkel AH (2009). Physiological and pharmacological roles of ABCG2 (BCRP): recent findings in Abcg2 knockout mice. Adv Drug Delivery Rev.

[39] Desuzinges-Mandon E, Arnaud O, Martinez L, Huche F, Di Pietro A, Falson P (2010). ABCG2 transports and transfers heme to albumin through its large extracellular loop. J Biol Chem.

[40] Kobuchi H, Moriya K, Ogino T, Fujita H, Inoue K, Shuin T (2012). Mitochondrial localization of ABC transporter ABCG2 and its function in 5-aminolevulinic acid-mediated protoporphyrin IX accumulation. PLoS ONE.

[41] Nakayama A, Matsuo H, Takada T, Ichida K, Nakamura T, Ikebuchi Y (2011). Abcg2 is a high-capacity urate transporter and its genetic impairment increases serum uric acid levels in humans. Nucleoside Nucleotides Nucleic Acids.

[42] Komiyama K, Iwasaki K, Miura M, Yamamoto H, Nozawa Y, Umezawa I (1985). Mechanism of action of antitumor antibiotic stubomycin. J Antibiot (Tokyo).

[43] Komiyama K, Edanami K, Tanoh A, Yamamoto H, Umezawa I (1983). Studies on the biological activity of stubomycin. J Antibiot (Tokyo).

[44] Sievers F, Wilm A, Dineen D, Gibson TJ, Karplus K, Li W (2011). Fast, scalable generation of high-quality protein multiple sequence alignments using Clustal Omega. Mol Syst Biol.

[45] Goujon M, McWilliam H, Li W, Valentin F, Squizzato S, Paern J (2010). A new bioinformatics analysis tools framework at EMBL-EBI. Nucleic Acids Res..

[46] Bairoch A, Apweiler R, Wu CH, Barker WC, Boeckmann B, Ferro S (2005). The universal protein resource (UniProt). Nucleic Acids Res..

[47] Kanai R, Ogawa H, Vilsen B, Cornelius F, Toyoshima C (2013). Crystal structure of a Na + -bound Na + , K + -ATPase preceding the E1P state. Nature.

[48] Trott O, Olson AJ (2009). AutoDock Vina: improving the speed and accuracy of docking with a new scoring function, efficient optimization, and multithreading. J Comput Chem.

[49] Irwin JJ, Shoichet BK (2005). ZINC—a free database of commercially available compounds for virtual screening. J Chem Inf Model.

[50] Lipinski CA (2004). Lead- and drug-like compounds: the rule-of-five revolution. Drug Discov Today Technol.

[51] Lipinski CA, Lombardo F, Dominy BW, Feeney PJ (1997). Experimental and computational approaches to estimate solubility and permeability in drug discovery and development settings. Adv Drug Deliv Rev.

[52] Suite S-MDD (2015). QikProp.

[53] Liu J, Hu Y, Waller DL, Wang JF, Liu QS (2012). Natural products as kinase inhibitors. Nat Prod Rep.

[54] Jorda R, Paruch K, Krystof V (2012). Cyclin-dependent kinase inhibitors inspired by roscovitine: purine bioisosteres. Curr Pharm Des.

[55] Khalil HS, Mitey V, Vlaykoya T, Cayicchi L, Zhelev N (2015). Discovery and development of Seliciclib. How systems biology approaches can lead to better drug performance. J Biotechnol.

[56] Maki N, Moitra K, Silver C, Ghosh P, Chattopadhyay A, Dey S (2006). Modulator-induced interference in functional cross talk between the substrate and the ATP sites of human P-glycoprotein. Biochemistry.

[57] Seelig A, Blatter XL, Wohnsland F (2000). Substrate recognition by P-glycoprotein and the multidrug resistance-associated protein. Int J Clin Pharmacol Ther.

[58] Seelig A (1998). A general pattern for substrate recognition by P-glycoprotein. Eur J Biochem.

[59] Priebe W, Perez-Soler R (1993). Design and tumor targeting of anthracyclines able to overcome multidrug resistance: a double-advantage approach. Pharmacol Ther.

[60] Mazel M, Clair P, Rousselle C, Vidal P, Scherrmann JM, Mathieu D (2001). Doxorubicin-peptide conjugates overcome multidrug resistance. Anticancer Drugs.

[61] Holmes AR, Keniya MV, Ivnitski-Steele I, Monk BC, Lamping E, Sklar LA (2012). The monoamine oxidase A inhibitor clorgyline is a broad-spectrum inhibitor of fungal ABC and MFS transporter efflux pump activities which reverses the azole resistance of Candida albicans and Candida glabrata clinical isolates. Antimicrob Agents Chemother.

[62] Schuetzer-Muehlbauer M, Willinger B, Egner R, Ecker G, Kuchler K (2003). Reversal of antifungal resistance mediated by ABC efflux pumps from Candida albicans functionally expressed in yeast. Int J Antimicrob Agents.

[63] Huang S, Cao YY, Dai BD, Sun XR, Zhu ZY, Cao YB (2008). In vitro synergism of fluconazole and baicalein against clinical isolates of Candida albicans resistant to fluconazole. Biol Pharm Bull.

[64] Tsuruo T, Iida H, Tsukagoshi S, Sakurai Y (1981). Overcoming of vincristine resistance in P388 leukemia in vivo and in vitro through enhanced cytotoxicity of vincristine and vinblastine by verapamil. Cancer Res.

[65] Teodori E, Dei S, Scapecchi S, Gualtieri F (2002). The medicinal chemistry of multidrug resistance (MDR) reversing drugs. Farmaco.

[66] Molnar J, Engi H, Hohmann J, Molnar P, Deli J, Wesolowska O (2010). Reversal of multidrug resitance by natural substances from plants. Curr Top Med Chem.

[67] McDevitt CA, Callaghan R (2007). How can we best use structural information on P-glycoprotein to design inhibitors?. Pharmacol Ther.

[68] Banoee M, Seif S, Nazari ZE, Jafari-Fesharaki P, Shahverdi HR, Moballegh A (2010). ZnO nanoparticles enhanced antibacterial activity of ciprofloxacin against Staphylococcus aureus and Escherichia coli. J Biomed Mater Res B Appl Biomater.

[69] Fiske CH, Subbarow Y (1925). The colorimetric determination of phosphorus. J Biol Chem.

[70] Yalaoui S, Zougbede S, Charrin S, Silvie O, Arduise C, Farhati K (2008). Hepatocyte permissiveness to Plasmodium infection is conveyed by a short and structurally conserved region of the CD81 large extracellular domain. PLoS Pathog.

[71] Silvie O, Greco C, Franetich JF, Dubart-Kupperschmitt A, Hannoun L, van Gemert GJ (2006). Expression of human CD81 differently affects host cell susceptibility to malaria sporozoites depending on the Plasmodium species. Cell Microbiol.

[72] Jacobson MP, Pincus DL, Rapp CS, Day TJF, Honig B, Shaw DE (2004). A hierarchical approach to all-atom protein loop prediction. Proteins Struct Funct Genet..

[73] Sastry GM, Adzhigirey M, Day T, Annabhimoju R, Sherman W (2013). Protein and ligand preparation: parameters, protocols, and influence on virtual screening enrichments. J Comput Aided Mol Des.

[74] Morris GM, Huey R, Lindstrom W, Sanner MF, Belew RK, Goodsell DS (2009). AutoDock4 and AutoDockTools4: automated docking with selective receptor flexibility. J Comput Chem.

[75] Shelley JC, Cholleti A, Frye LL, Greenwood JR, Timlin MR, Uchimaya M (2007). Epik: a software program for pK(a) prediction and protonation state generation for drug-like molecules. J Comput Aided Mol Des..

[76] Jorgensen WL, Maxwell DS, TiradoRives J (1996). Development and testing of the OPLS all-atom force field on conformational energetics and properties of organic liquids. J Am Chem Soc.

[77] Kaminski GA, Friesner RA, Tirado-Rives J, Jorgensen WL (2001). Evaluation and reparametrization of the OPLS-AA force field for proteins via comparison with accurate quantum chemical calculations on peptides. J Phys Chem B.

[78] O’Boyle NM, Banck M, James CA, Morley C, Vandermeersch T, Hutchison GR (2011). Open Babel: an open chemical toolbox. J Cheminf.

