# Supplementary material for: Two inhibitors of yeast plasma membrane ATPase 1 (ScPma1p): toward the development of novel antifungal therapies
Source: J Cheminform. 2018 Feb 20;10:6. doi: 10.1186/s13321-018-0261-3 (PMC5820243; doi:10.1186/s13321-018-0261-3)
Supplement: Supplementary file 1 — Additional file 1: Table S1. A list of additional antifungal compounds found in our whole-cell screen. Figure S1. IC50 curves for the cell-free, vesicle-based ScPma1p assays. Figure S2. IC50 curves for the whole-cell assays. Figure S3. Compound IC50 values against whole-cell ABC16-Monster yeast, with and without two distinct spiroindolone-binding-pocket ScPMA1 mutations (L290S and P399T). [file 13321_2018_261_MOESM1_ESM.docx]

# **Two Inhibitors of Yeast Plasma Membrane ATPase 1 (*Sc*Pma1p): Toward the Development of Novel Antifungal Therapies**

# Sabine Ottilie^1,11^, Gregory M. Goldgof^2,11^, Andrea L. Cheung^3^, Jennifer L. Walker^4^, Edgar Vigil^5^, Kenneth E. Allen^6^, Yevgeniya Antonova-Koch^7^, Carolyn W. Slayman^8^, Yo Suzuki^9^, Jacob D. Durrant^10,*^

^1^ Division of Host Pathogen Systems and Therapeutics, Department of Pediatrics, University of California, San Diego, School of Medicine, La Jolla, California 92093, United States. sottilie2@gmail.com

^2^ Division of Host Pathogen Systems and Therapeutics, Department of Pediatrics, University of California, San Diego, School of Medicine, La Jolla, California 92093, United States, and Department of Synthetic Biology and Bioenergy, J. Craig Venter Institute, La Jolla, California 92037, United States. ggoldgof@gmail.com

^3^ Division of Host Pathogen Systems and Therapeutics, Department of Pediatrics, University of California, San Diego, School of Medicine, La Jolla, California 92093, United States. andrealcheung@gmail.com

# ^4^ Department of Biological Sciences, University of Pittsburgh, Pittsburgh, Pennsylvania 15260, United States. jlwalker@pitt.edu

^5^ Division of Host Pathogen Systems and Therapeutics, Department of Pediatrics, University of California, San Diego, School of Medicine, La Jolla, California 92093, United States. elcibrian@gmail.com

^6^ Department of Genetics, Yale University School of Medicine, New Haven, Connecticut 06520, United States. kenneth.allen@yale.edu

^7^ Division of Host Pathogen Systems and Therapeutics, Department of Pediatrics, University of California, San Diego, School of Medicine, La Jolla, California 92093, United States. jenya.antonova@gmail.com

^8^ Department of Genetics, Yale University School of Medicine, New Haven, Connecticut 06520, United States. carolyn.slayman@yale.edu

^9^ Department of Synthetic Biology and Bioenergy, J. Craig Venter Institute, La Jolla, California 92037, United States. YSuzuki@jcvi.org

# ^10^ Department of Biological Sciences, University of Pittsburgh, Pittsburgh, Pennsylvania 15260, United States. [durrantj@pitt.edu](mailto:durrantj@pitt.edu)

^11^These two authors contributed equally to this work.

# ^*^ To whom correspondence should be addressed: durrantj@pitt.edu

#

# **Keywords**

Antifungal, PMA1, P-type ATPase, Computer-Aided Drug Discovery, Virtual Screening, *Saccharomyces cerevisiae*, In Vitro Evolution, Drug Resistance

| Compound | Percent Inhibition | Compound | Percent Inhibition |
| --- | --- | --- | --- |
|   NSC343256 (hitachimycin) | 101.0 |   NSC41098 | 99.9 |
|   NSC116644 | 99.7 |   NSC15784 | 99.5 |
|   NSC228150 | 99.4 |   NSC622689 | 99.4 |
|   NSC661221 | 99.2 |   NSC369066 | 99.2 |
|   NSC354844 | 99.2 |   NSC98363 | 99.1 |
|   NSC11668 | 99.1 |   NSC338106 | 99.0 |
|   NSC2805 | 98.9 |   NSC92937 | 98.9 |
|   NSC727038 | 98.9 |   NSC70931 | 98.8 |
|   NSC139021 | 98.7 |   NSC46492 | 98.7 |
|   NSC270916 | 98.4 |   NSC96996 | 98.3 |
|   NSC33005 | 98.3 |   NSC7420 | 98.2 |
|   NSC636718 | 98.1 |   NSC645330 | 98.1 |
|   NSC26112 | 98.0 |   NSC40749 | 98.0 |
|   NSC637827 | 97.9 |   NSC13151 | 97.9 |
|   NSC105781 | 97.9 |   NSC80997 | 97.7 |
|   NSC10091 | 97.5 |   NSC58904 | 97.5 |
|   NSC11881 | 97.4 |   NSC48617 | 97.3 |
|   NSC83950 | 97.2 |   NSC281383 | 97.1 |

**Table S1.** The 36 NCI IV compounds that inhibited the growth of ABC_16_-Monster by at least 97% at 100 μM.

**
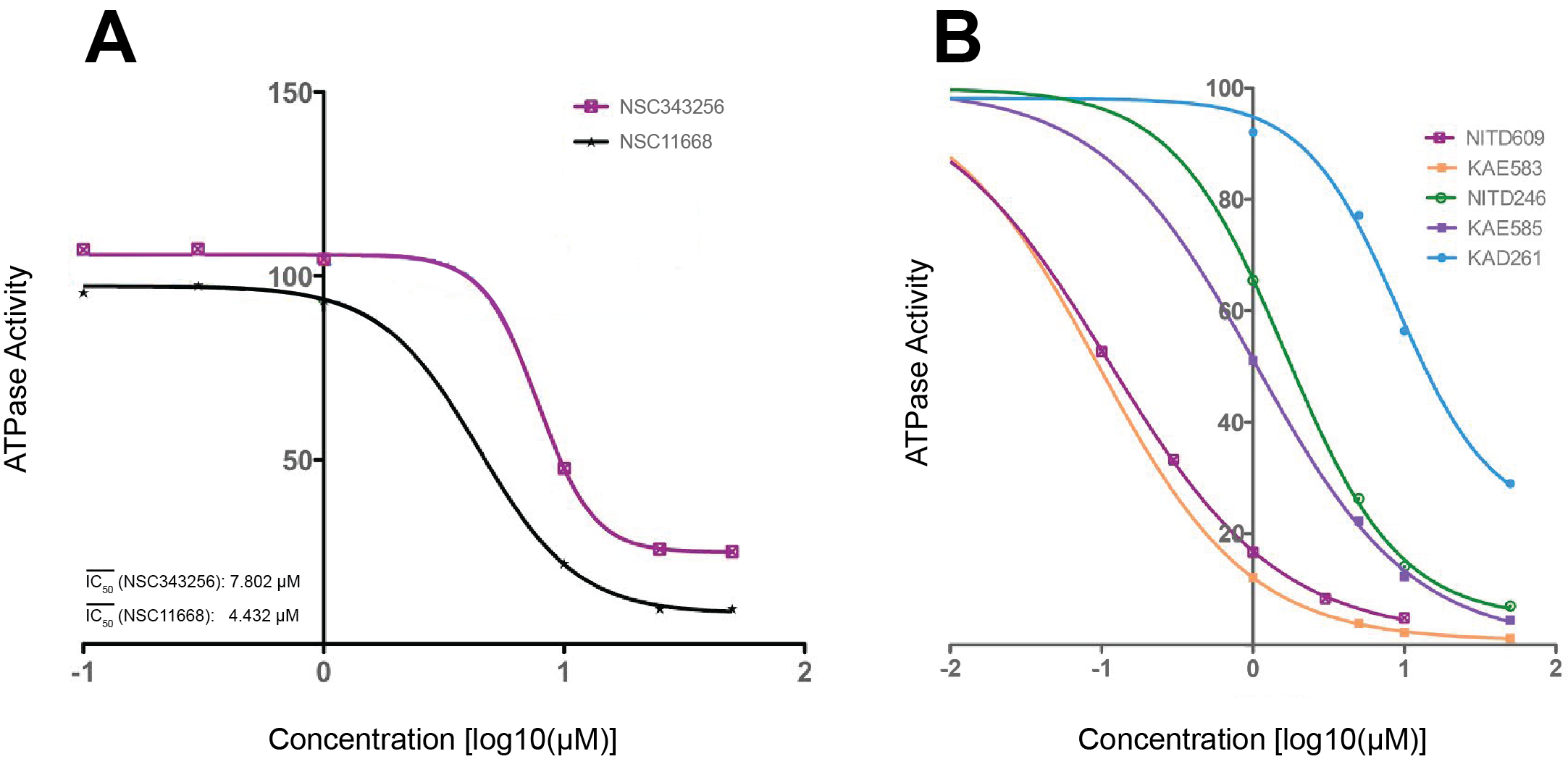
**

**Figure S1. IC_50_ curves for the cell-free, vesicle-based *Sc*PMA1 assays.** A) NSC11668 and hitachimycin activity. The mean IC_50_ values are inset. Each curve was calculated from experiments performed in duplicate. B) The activities of five known *Sc*PMA1 inhibitors (positive controls). Data taken from ref. (1). IC_50_(NITD609): 0.112 µM; IC_50_(KAE583): 0.095 µM; IC_50_(NITD246): 1.646 µM; IC_50_(KAE585): 1.047 µM; IC_50_(KAD261): 9.086 µM.

**
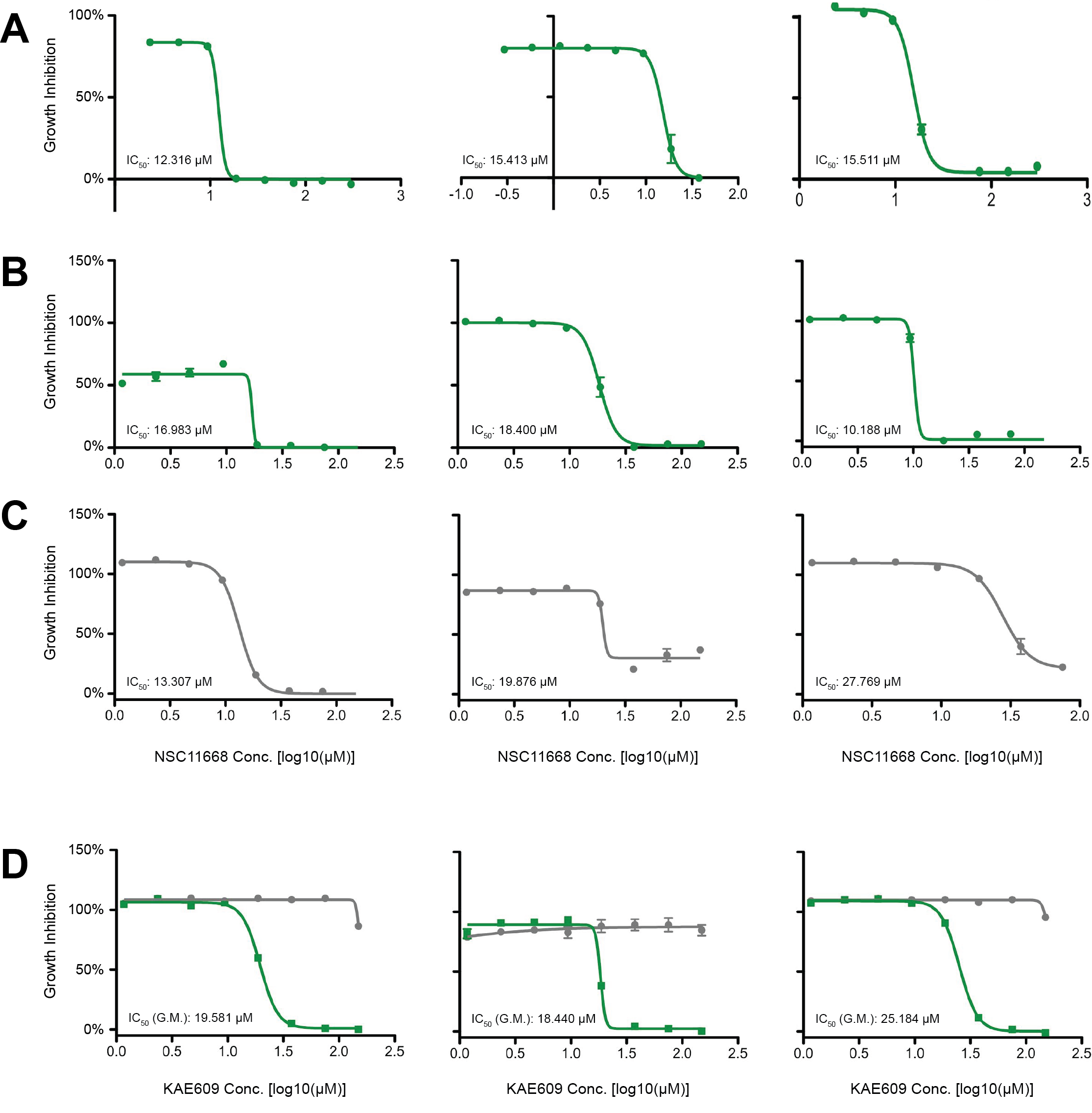
**

**Figure S2. IC_50_ curves for whole-cell assays.** Curves shown in green and gray correspond to the Green-Monster (*GM*) and wild-type (*wt*) yeast strains, respectively. Individual data points were derived from experiments performed in duplicate or triplicate. To verify the consistency of our assay, we calculated multiple *GM* and *wt* IC_50_ curves, each curve from experiments performed on a different day. The calculated IC_50_ values (inset) were consistently similar. The IC_50_ values reported in the main text are the average values over all days. Rows A and B) NSC11668, the more promising lead, against *GM*. Row C) NSC11668 against *wt*. Row D) KAE609, serving as a positive control, against both *GM* and *wt*. The inset IC_50_ values are for *GM*. The *wt* IC_50_ values are >150 µM.

**
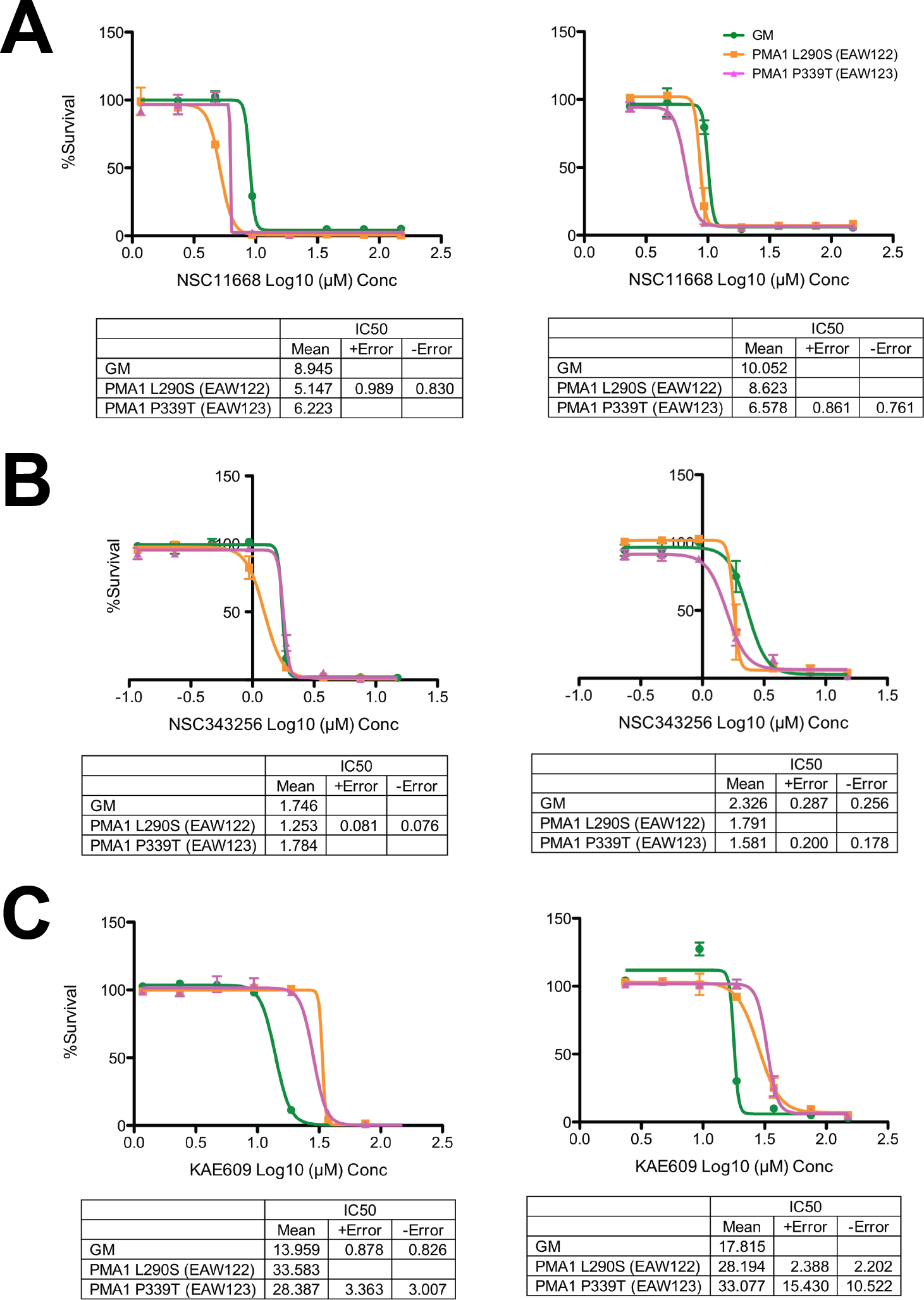
**

**Figure S3. Compound IC_50_ values against whole-cell ABC_16_-Monster yeast, with and without two distinct spiroindolone-binding-pocket *Sc*PMA1 mutations: L290S and P399T.** In all graphs, the ABC_16_-Monster curves are shown in green, and the L290S and P399T curves are shown in orange and purple, respectively. Each experiment was performed in duplicate (left and right side, respectively). A) NSC11668 and B) NSC343256 (hitachimycin) inhibition are unaffected by the altered spiroindolone-binding pocket. C) KAE609 inhibition (positive control) is much reduced against strains with altered spiroindolone-binding pockets, as expected.

**References**

1. Goldgof GM, Durrant JD, Ottilie S, Vigil E, Allen KE, Gunawan F, et al. Comparative chemical genomics reveal that the spiroindolone antimalarial KAE609 (Cipargamin) is a P-type ATPase inhibitor. Sci Rep. 2016;6.
